# Development and Validation of the Vision-Related Dizziness Questionnaire

**DOI:** 10.3389/fneur.2018.00379

**Published:** 2018-05-29

**Authors:** Deborah Armstrong, Alison J. Alderson, Christopher J. Davey, David B. Elliott

**Affiliations:** School of Optometry and Vision Science, University of Bradford, Bradford, United Kingdom

**Keywords:** vision-related dizziness, dizziness, patient-reported outcome measure, questionnaire, Rasch analysis

## Abstract

**Purpose:**

To develop and validate the first patient-reported outcome measure (PROM) to quantify vision-related dizziness. Dizziness is a common, multifactorial syndrome that causes reductions in quality of life and is a major risk factor for falls, but the role of vision is not well understood.

**Methods:**

Potential domains and items were identified by literature review and discussions with experts and patients to form a pilot PROM, which was completed by 335 patients with dizziness. Rasch analysis was used to determine the items with good psychometric properties to include in a final PROM, to check undimensionality, differential item functioning, and to convert ordinal questionnaire data into continuous interval data. Validation of the final 25-item instrument was determined by its convergent validity, patient, and item-separation reliability and unidimensionality using data from 223 patients plus test–retest repeatability from 79 patients.

**Results:**

120 items were originally identified, then subsequently reduced to 46 to form a pilot PROM. Rasch analysis was used to reduce the number of items to 25 to produce the vision-related dizziness or VRD-25. Two subscales of VRD-12-frequency and VRD-13-severity were shown to be unidimensional, with good psychometric properties. Convergent validity was shown by moderately good correlations with the Dizziness Handicap Inventory (*r* = 0.75) and good test–retest repeatability with intra-class correlation coefficients of 0.88.

**Conclusion:**

VRD-25 is the only PROM developed to date to assess vision-related dizziness. It has been developed using Rasch analysis and provides a PROM for this under-researched area and for clinical trials of interventions to reduce vision-related dizziness.

## Introduction

Dizziness is common in older people (~30% in patients over 65 years of age) (1), can have significant negative effects on quality of life (2, 3), and is a major risk factor for falls (4). Although vestibular disease and central vascular disease are the most commonly reported diagnoses in secondary/tertiary care (1, 5) and primary care (1), respectively, the prevalence of specific causes of dizziness varies hugely (1, 6) and dizziness is a multifactorial geriatric syndrome (1–3, 6) like falls. Causes include vestibular disease, psychiatric disorders, vascular disease including hypotension, polypharmacy, medication side effects, Parkinson’s disease, and visual impairment. There are several subcategories (7) of dizziness: disequilibrium, light-headedness, pre-syncope, and vertigo and each may be related to different diagnoses. Visual impairment (1) and changes in refractive correction (8) likely have a role in the dizziness subcategories of vertigo (the feeling that either the individual or their surroundings are spinning) *via* the vestibulo-ocular reflex and disequilibrium (the feeling that an individual cannot keep their balance when they are standing still) *via* the influence of visual function on postural stability (9).

Although the literature investigating the link between vision and dizziness is relatively small, the authors of a systematic review concluded that large-sample, well-designed epidemiological studies found that self-reported visual impairment was a significant independent predictor of dizziness (10). In addition, a recent cohort study found that dizziness reduced following first-eye cataract surgery by an amount linked to the improvement in best eye visual acuity, but was increased by changes in oblique astigmatic refractive correction (11). Finally, patients with Visual Vertigo report dizziness that is triggered by visual motion such as when walking in supermarket aisles, driving, and watching moving scenes. This condition is thought to be due to overreliance on visual control after vestibular disease or other insult (12, 13).

There are currently no patient-reported outcome measures (PROMs) that assess vision-related dizziness (3). The Visual Vertigo Analog Scale (14) is a nine-item visual analog scale to assess the intensity of symptoms for patients with Visual Vertigo which was developed using classical test theory. It is restricted to assessment of the one condition of Visual Vertigo. Most of the commonly used dizziness PROMs, such as the current standard, the Dizziness Handicap Inventory (DHI), were developed for patients with vestibular disease (15). In addition, the great majority of PROMs that target dizziness used traditional classical test theory questionnaire development methods (3, 15) and a systematic review concluded that there were no validated PROMs for the study of age-related vestibular loss in clinical trials (15).

Preferred for the development of PROMs are the psychometric methods of item response theory that includes Rasch analysis (16–20). These have many advantages over classical test theory such as the conversion of ordinal data from questionnaire responses into continuous interval data, allowing linear measurement and determining dimensionality (16, 18). Indeed, traditionally developed PROMs have been re-engineered and scored using Rasch analysis when assessing vision-related quality of life (19, 20). Rasch analysis has been used to develop a short-form (13 items) of the 25-item DHI (21) and we have used this PROM in a study of dizziness before and after cataract surgery (11). However, it was developed to assess patients with vestibular disease, with 6 of 13 items being directed at symptoms from such patients (items relate to dizziness when getting in and out of bed, turning over in bed, bending over, quick head movements, looking up, and walking in the dark) with just three items being related to vision in some way (dizziness when reading, walking down the aisle of a supermarket, and walking on the sidewalk, the latter two being well-known triggers for Visual Vertigo) and is limited in its assessment of vision-related dizziness.

The aim of this study was to develop and validate using Rasch analysis the first PROM to quantify vision-related dizziness. This would provide a PROM for clinical research in the under-researched area of vision-related dizziness and for clinical trials of vision and refractive interventions aimed to reduce dizziness.

## Materials and Methods

### PROM Development

Patient-reported outcome measure development and validation generally followed the recommendations by Pesudovs et al. (16). This study was carried out in accordance with the recommendations of the Research Ethics Committees of both the University of Bradford and the UK National Health Service (UK EC1843 and IRAS 180272). The protocol was approved by these committees. All subjects gave written informed consent in accordance with the Declaration of Helsinki.

### Domain and Item identification

Patient-reported outcome measures relating (separately) to visual disability, vision-related quality of life, and dizziness were identified from a comprehensive search of the literature and were examined to identify domains and items that could be related to vision-related dizziness. The structure of the Rasch-developed visual symptom PROM, Quality of Vision (22), with its three subscales of the “frequency,” “severity,” and “bothersomeness” of symptoms, was incorporated into the item identification process. We previously found that a “frequency” based questionnaire developed to assess spectacle adaptation symptoms (which included dizziness), appeared to be limited by a lack of assessment of the severity/bothersomeness of symptoms (23). Response categories of “so severe/bothersome I have reduced doing this” and “so severe/bothersome I have stopped doing this” provided assessment of the activity limitation aspects of dizziness. The domains and items identified by the literature search and further identification of other domains and items were discussed with nine experienced clinicians and structured interviews with patients who self-reported problems due to dizziness. The clinicians included two consultant geriatricians (in the UK, a consultant physician typically has at least 10 years post registration experience and is similar to a US attending physician) and a specialist physiotherapist with 10+years of experience, a consultant ophthalmologist, two optometrists (authors David B. Elliott and Alison J. Alderson, with 20+years of clinical experience), two ENT consultants, and a hospital specialist audiologist with 20+years of experience. The nine patients were recruited from the staff of the University *via* an email request and patients from local vision and falls clinics and included patients with self-reported vision-related dizziness linked to age-related cataract (two) multifocal spectacle lens use (two), large spectacle power changes (one), one patient with Visual Vertigo and three patients with vestibular disease who helped differentiate between dizziness linked to vestibular disease and vision-related issues. Patients were recruited and interviewed until saturation of themes had been reached. They were asked about the important quality of life issues that were affected by their dizziness.

### Item Reduction

Items deemed to be irrelevant, duplicate, or unhelpful were then discarded by a three-step process. First, by separate consultation with the clinicians described above. Second, by a focus group of six of those clinicians. Third, by a focus group of four patients (age range 35–79 years, two females) who were recruited *via* an email request from the staff of the University and patients at the University Eye Clinic and reported vision-related dizziness (two diagnosed with Visual Vertigo, two with a history of dizziness due to multifocal spectacles).

### Cognitive Interviews

After item reduction, the patient focus group provided cognitive interview information by discussing the readability and ease of comprehension of the items and response categories. This resulted in appropriate wording changes to some questions and the addition of clarification of what was meant by each response category. For example, the category of “Very Often” could mean different frequencies to different people, so the focus group suggested that adding “(e.g., 2–6 times per week)” would make it easier for participants to understand what was meant.

### Pilot Questionnaire

As the diagnosis of dizziness is multifactorial and challenging and many patients are unaware of whether vision is part of the etiology of their dizziness (1), the inclusion criteria for completion of the pilot questionnaire included any patient with self-reported dizziness who was over 18 years and had suffered from dizziness in the past month. An electronic version of the questionnaire was created using Wufoo (http://www.wufoo.com) and indicated that a questionnaire was being developed to quantify vision-related dizziness. Patients gave their consent by submitting a completed online questionnaire. The research was publicized *via* e-newsletters and social media to international dizziness-related support groups (e.g., the Vestibular Disorders Association, Vertigo & Meniere’s disease support group), national support centers for older people (e.g., Women’s Institute of the UK and Canada; Age UK), and a wide range of regional UK older peoples’ forums and support groups. Paper copies of the pilot instrument were made available to patients with dizziness in regional Falls, Vestibular Diseases and Audiology Clinics, and informed consent was gained from patients who completed the paper version of the questionnaire. The pilot questionnaire was available for completion on the Wufoo site between April 4, 2016 and June 21, 2016. The minimum target sample size was 250 (24). Additional information collected included respondent age, sex, cause of dizziness, and whether they had fallen in the last 6 months.

### Data Analysis of Pilot Questionnaire

Rasch analysis (Winsteps version 3.91.0; Winsteps, Chicago, IL, USA) using an Andrich rating scale model assessed the response categories and individual person data. Individual items from the pilot data were examined in terms of their fit to the Rasch model of Infit and Outfit [values outside the range of 0.60–1.40; this is slightly more lenient than the 0.70–1.30 suggested by Pesudovs et al. (16) and follows Wright and Linacre] (25), normality of the distribution including ceiling and floor effects, proportion of missing data (>33%) (26), and the distance of the item mean response to the mean participant response (16). Poorly fitting items were removed iteratively with the model being reanalyzed after each item elimination. If the fit of the model was negatively affected by the removal of an item, that item was re-introduced. The process was repeated until all items fitted well and the content of the final instrument, the vision-related dizziness (VRD-25) PROM, was determined. Unidimensionality of VRD-25 was checked with principal components analysis. Any subscales identified by the principal components analysis were then reanalyzed using Rasch analysis to ensure robust psychometric properties. Dimensionality of VRD-25 was assessed using principal components analysis to test for unidimensionality and differential item functioning (DIF) was used to assess whether different groups (such as younger vs. older participants, males vs. females) answered the questions differently. A significant difference was taken as the Rasch–Welch DIF contrast being >0.5 logits and the *t* value being ≥ ±2 (27).

### Assessment of VRD Performance

Electronic versions of the VRD-25 and the DHI, were created using Wufoo (http://www.wufoo.com) and publicized *via* the same e-newsletters and social media used previously. Respondents were asked to complete both questionnaires and they were available on the Wufoo site between November 1, 2016 and February 22, 2017. Performance of the VRD-25 was assessed using convergent validity, person, and item-separation reliability, test–retest agreement, dimensionality using principal components analysis, and DIF using Rasch analysis.

Convergent validity was assessed by the amount of correlation with a related measure, the most commonly used dizziness PROM, the DHI (15). Acceptable performance would be a correlation coefficient between VRD and DHI between 0.30 and 0.90 (16). This is the first step in evaluating validity of VRD and future studies may assess other aspects of validity.

Discriminative ability was assessed using Rasch person and item-separation reliabilities. These measure how well the items of the instrument differentiate between people with different levels of dizziness. Good separation indices indicate that the instrument can discriminate between people with different levels of dizziness. For example, a person-separation index of >2.0 indicates that the instrument can discriminate between people with high and low symptom levels and good performance would be reliability coefficients ≥0.80 (16).

Test–retest reliability of VRD was determined by re-administering VRD (and DHI for comparison) after 2–4 weeks to those participants who had consented. Good performance would be a test–retest intra-class correlation coefficient ≥0.80 (16). Correlation analyses were performed using SPSS statistics for Windows (version 23.0; IBM Corp., Armonk, NY, USA).

## Results

**Figure 1:**
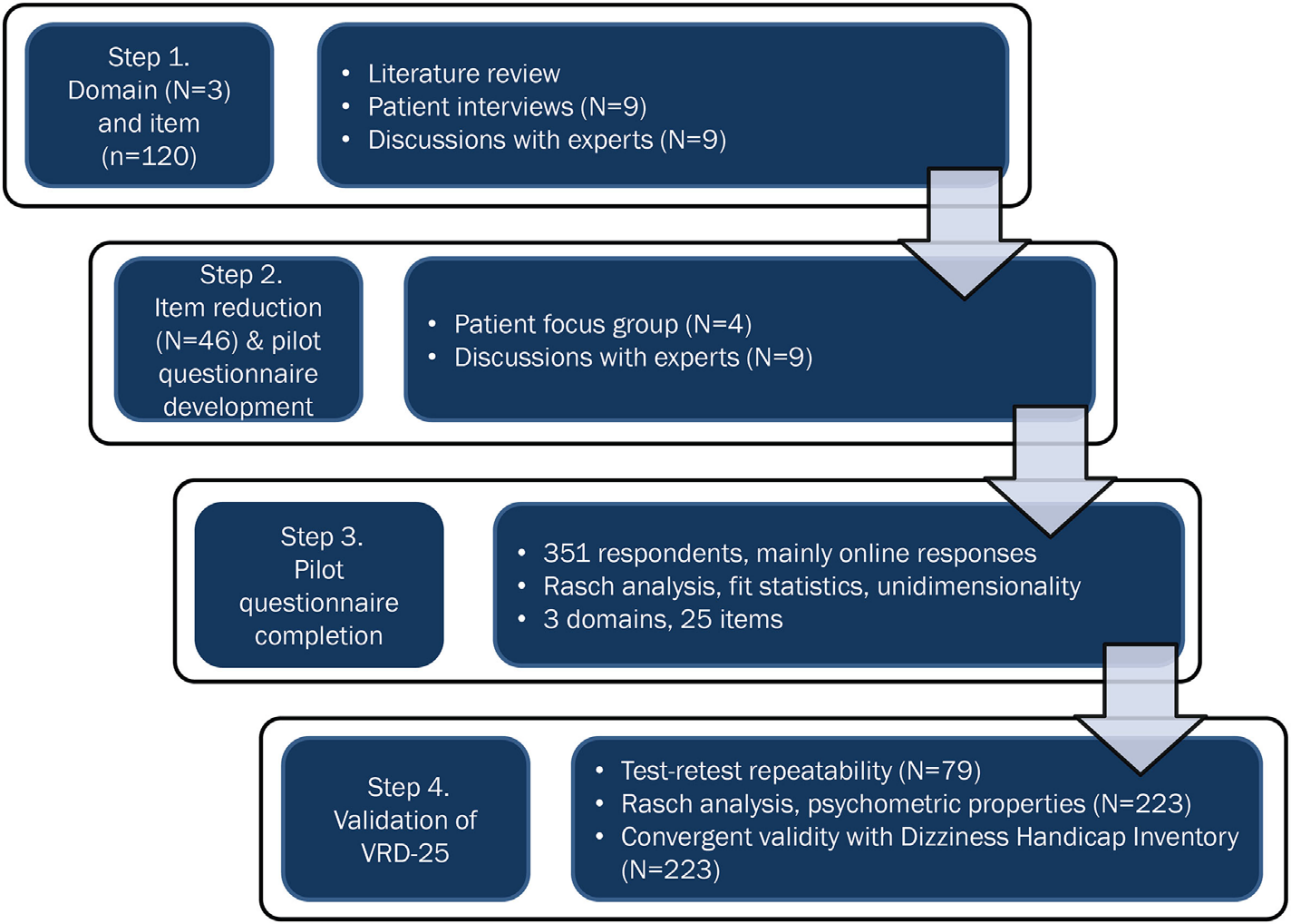
A flowchart showing the development and validation process for the 25-item vision-related dizziness (VRD-25) patient-reported outcome measure.

The development process is summarized in Figure 1.

### Domain and Item Identification

One hundred twenty items under three domains of symptoms, activity limitation, and psychosocial issues were identified. Items included the frequency and severity of dizziness related to reading, walking alongside a busy road, down the aisle of a supermarket, on sloping surfaces, up or down stairs, moving around the home, stepping onto an escalator, watching moving scenes on TV, watching a scrolling computer screen, when driving a car, and looking from a height; plus psychosocial issues due to dizziness of difficulties concentrating, feeling confused, anxious or upset, people thinking you are intoxicated and being afraid to leave the home.

### Item Reduction

Questions that related to “light-headedness” (most often used to describe symptoms associated with postural hypotension) were removed from the list of possible items, as the results of a systematic review of the link between vision and dizziness suggested these were not relevant to vision-related dizziness (10). Initially, items included three versions relating to the frequency, severity, and bothersomeness of symptoms (22). However, the clinicians and patient focus group felt that if a patient found their dizziness to be “bothersome,” they would rate it as “severe” and they thought the latter term was more understandable so that “bothersome” response categories were removed. In addition, the original Quality of Vision instrument reported the lowest person-separation reliability and construct validity for the bothersomeness scale (22). A five-point Likert-type response scale for responses was used to minimize respondent burden while maximizing measurement of the construct (28). The resulting 46-item instrument, including three domains of symptoms (17 items), activity limitation (17 items), and psychosocial issues (12 items), formed the pilot questionnaire.

### Pilot Study Questionnaire

The pilot instrument was completed by 351 participants with a mean age of 57 ± 14, range 20–94 years; 79% were female; 95% completed online; 75% were from North America, 21% from Europe, and 4% other; the most common self-reported causes of participants’ dizziness were Vestibular (including Ménière’s Disease, Vertigo, and Labyrinthitis) 58%, unknown 26%, visual 5%, and other 11%; 38% had fallen in the last 6 months.

**Figure 2:**
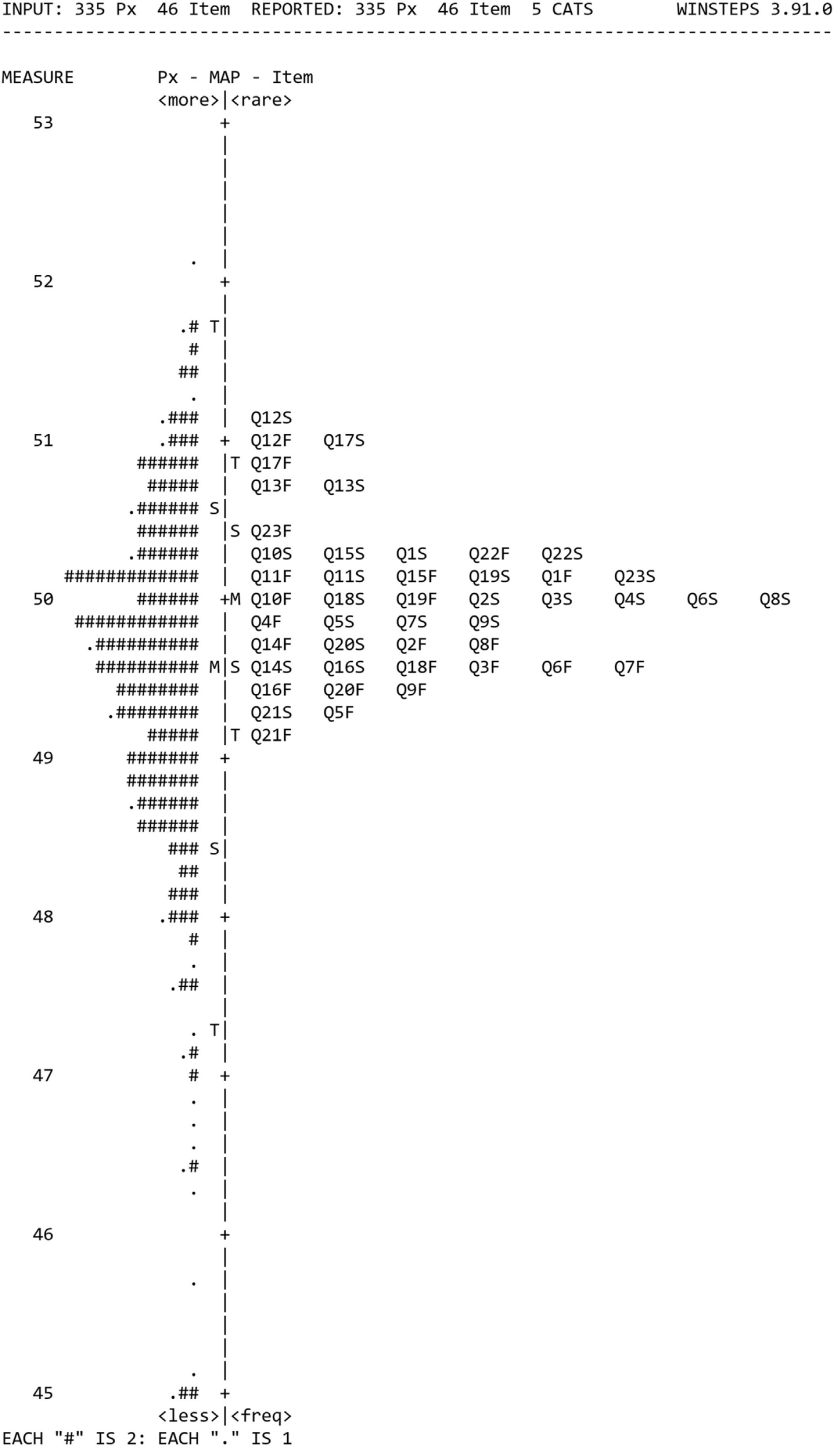
The Rasch person-item map from the 335 responses to the pilot questionnaire, with mean positions of items on the right and respondents on the left.

Each item and person’s responses were examined and 16 sets of individual respondent data were discarded as they were incomplete [>33% missing responses (26)] or reported not being dizzy in the previous month. All items provided less than 33% missing responses, and all were included in the analyses. Category probability curves were generated from the remaining sample of 335 and suggested no redundancy of response categories and only minor differences between response categories *3 and 4 which were not alleviated by* collapsing the two categories so that they were retained. The Rasch person-item map is shown in Figure 2. It shows the average positions of items on the right as numbers from Q1 to Q23 for both F (frequency) and S (Severity) items, with the items at the top being more rarely endorsed as they were only responded to positively by people who had high levels of dizziness. The respondents are on the left of the map with a # representing two respondents. Respondents at the top have more frequent and severe symptoms than those at the bottom. The person-separation and item-separation reliabilities were 0.94 and 0.98, respectively. Using the procedure described in the methods, twenty-one (46%) poorly fitting items were iteratively removed, to leave VRD consisting of 25 items. VRD-25 is shown in Presentation S1 in Supplementary Material.

### VRD-25 Validation

Differential item functioning (a test of item bias) showed no significant differences for age (above and below the median age of 57). Slight DIF differences were found between male and female respondents for three unconnected items and were not deemed sufficiently important to remove the item. The VRD-25 provided an excellent person-separation reliability of 0.94 and item-separation reliability of 0.98, which are well above the level required for good performance of ≥0.80 (16).

Principal components analysis indicated that the data were not unidimensional, with 57% of the raw variance explained by the measure and the eigenvalue of the first contrast being 3.2 and above the cutoff value of 2.0 (27). Two components were indicated by principal components analysis and these clearly split into the items that related to the “severity” of the dizziness and its “frequency.” These two subscales were then assessed using Rasch analysis and principal components analysis. Both subscales of “frequency” (termed VRD-12f) and “severity” (VRD-13s) items were found to be unidimensional (VRD-12f 58%, VRD-13s 59% raw variance explained, all eigenvalues below 1.95). Rasch indices remained very good for all items (infit and outfit values within 0.60–1.40), person-separation reliabilities (VRD-12f: 0.88; VRD-13s: 0.90), and item-separation reliabilities (VRD-12f: 0.96; VRD-13s: 0.98).

**Figure 3:**
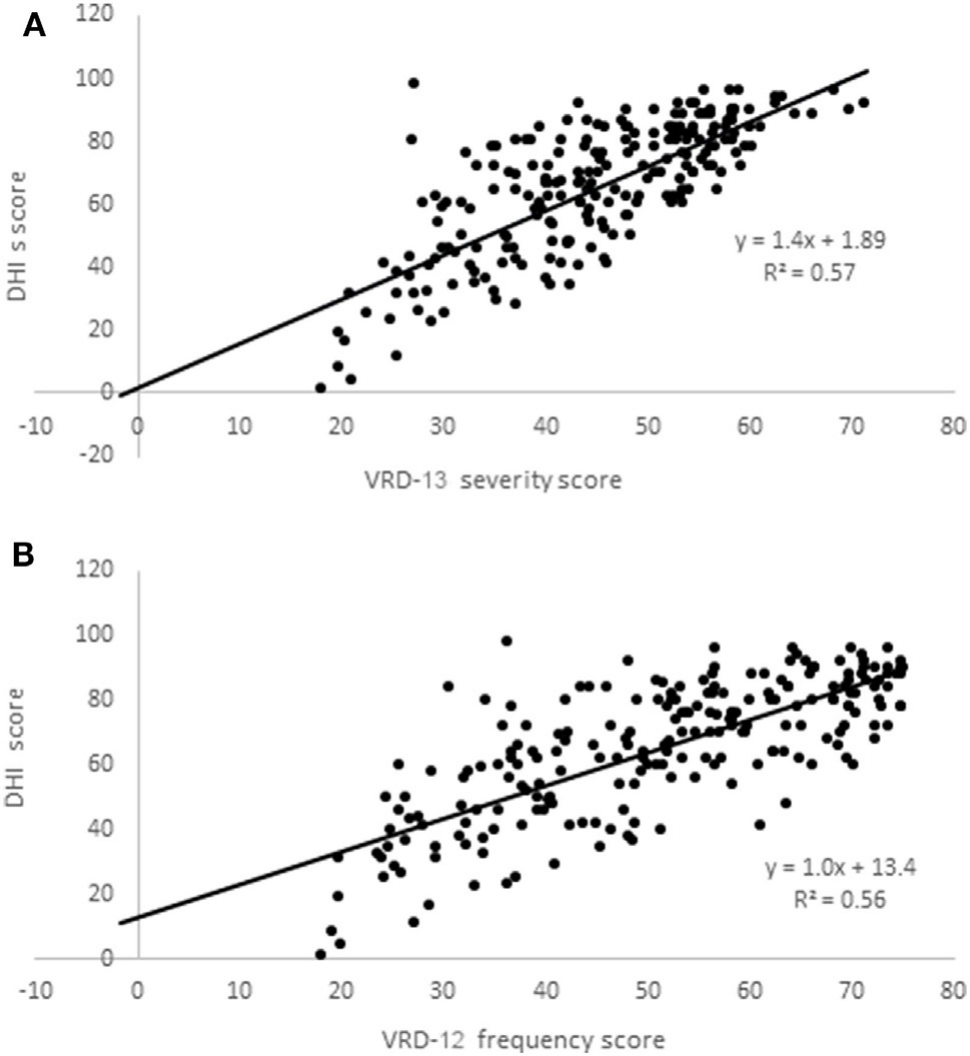
Scatterplots of Vision-related dizziness-severity (VRD-13s); **(A)** and VRD-frequency (VRD-12f); **(B)** scores vs. dizziness handicap inventory (DHI) scores.

VRD-25 and DHI were completed by a further 223 participants (mean age 48, SD 12 years; 83% female; 100% completed online; 56% from North America, 29% from Europe including 26% UK, 7% Oceania, and 8% other). VRD-12f and VRD-13s data were normally distributed (Kolmogorov–Smirnov, *p* > 0.10) but DHI data were not (*p* = 0.021). Rasch analysis indicated the following person-separation reliabilities (VRD-12f: 0.88; VRD-13s: 0.90) and item-separation reliabilities (VRD-12f: 0.97; VRD-13s: 0.96), with other psychometric properties similar to the pilot instrument data. Scatterplots of VRD-13s and VRD-12f vs. DHI are shown in Figure 3, showing Spearman correlation coefficients between the two of 0.75 (VRD-12f vs. DHI) and 0.76 (VRD-13s vs. DHI).

**Figure 4:**
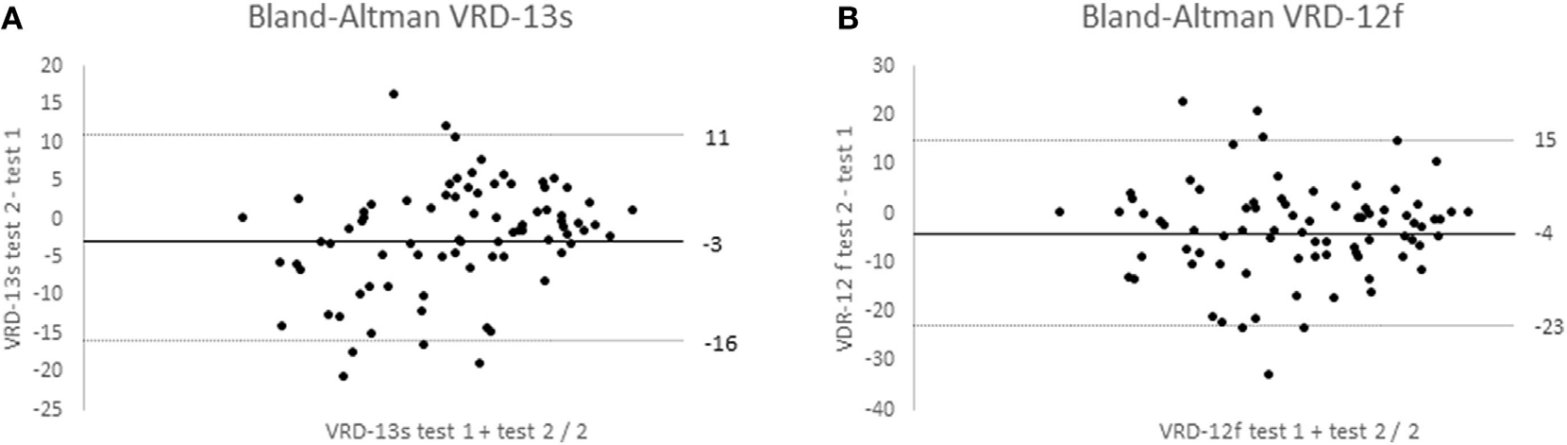
Bland–Altman plots of Vision-related dizziness-severity (VRD-13s); **(A)** and VRD-frequency (VRD-12f); **(B)** showing mean test–retest differences and 95% confidence limits for 70 participants.

VRD-25 and DHI repeatability data was obtained from 82 participants (mean age 51, SD 11 years; 90% female; 100% completed online; 54% from North America, 27% from Europe including 21% UK, 11% Oceania, and 8% other) and data from 3 participants were discarded as they showed major changes in dizziness score (>33 on the 0–100 scales). The Bland–Altman 95% repeatability values were ±13 (DHI), ±19 logits (VRD-12f), and ±14 logits (VRD-13s) (Figure 4). To allow comparison with earlier reports, intra-class correlation coefficients between test and retest data were calculated and found to be 0.92 (DHI), 0.88 (VRD-12f), and 0.88 (VRD-13s). The mean time taken to complete the PROM was 6 (range 3–10) minutes.

## Discussion

As expected, the VRD-25-item instrument was found to be multidimensional with two clear subscales, one containing items related to the frequency of vision-related dizziness issues and the other related to their severity. These two subscales were found to be unidimensional, which is a prerequisite before item responses can be summed to provide a total score (17, 28). Our previous experience (23) and that of others (22) is that the assessment of symptoms appears to need an assessment of both their frequency and severity/bothersomeness. The VRD-25 subscales of frequency (VRD-12f) and severity (VRD-13s) were individually shown to have excellent person-separation reliability (>0.87) and item-separation reliability (>0.89) by Rasch analysis and well above the recommended level for good performance of 0.80 (16). Convergent validity was determined by the association with the DHI and was found to be within the acceptable performance band of between 0.30 and 0.90 (16) (VRD-12f, *r* = 0.70; VRD-13s, *r* = 0.73). Test–retest repeatability of the VRD-25 was well above the good performance level of test–retest intra-class correlation coefficients (0.80) at 0.88 and 95% confidence limits of agreement were ±14 (VRD-13s) and ±19 logits (VRD-12f) and similar to the repeatability of the DHI (*r* = 0.92, ±13, respectively). The DHI repeatability data are similar to that reported by the developers of the instrument with morning–afternoon test–retest data (from which you might expect reduced variability compared to a 2-week test–retest period) from 14 participants of ±18 limits of agreement and a test–retest correlation coefficient of 0.97.

A limitation of the study is that the participants who completed the pilot and final PROMs may not be representative of patients with vision-related dizziness. Indeed, 58% reported that the principal cause of their dizziness was Vestibular disease (including Ménière’s Disease, Vertigo, and Labyrinthitis) and only 5% reported their dizziness was mainly due to a vision problem. However, dizziness is multifactorial (1–3, 6) and vision plays a greater role in controlling balance when the vestibular system is impaired (29), so that any impairment in vision in patients with vestibular disease is likely to lead to increased dizziness. This is highlighted by the condition “Visual Vertigo” which is thought to be due to overreliance on visual control after vestibular disease or other insult (12, 13). In addition, the prevalence of vision-related dizziness may be underestimated (for example, patients may believe that their dizziness is due to a vestibular problem yet have Visual Vertigo) and there is little high quality epidemiological data investigating the link between visual impairment and dizziness (10). It may be that once more is known about the visual contribution to dizziness, an improved vision-related dizziness PROM can be developed. The high percentage of respondents being female (79%) may be because dizziness is more common in women and particularly older women (2), who constituted the majority of our respondents. It may be that women are also more likely to be members of support groups such as those we targeted like the VDA, Pensioner forums, and of course, the Women’s Institute.

In summary, VRD-25 is the only PROM developed to date to assess vision-related dizziness. It has been developed using Rasch analysis and the two subscales of VRD-12 (frequency) and VRD-13 (severity) provided good psychometric properties, convergent validity, and test–retest agreement. VRD-25 can be used to develop research into the link between dizziness and visual impairment and refractive correction and provides a PROM for clinical trials of vision and refractive interventions that could reduce dizziness.

## Ethics Statement

This study was carried out in accordance with the recommendations of the Research Ethics Committees of both the University of Bradford and the UK National Health Service (UK EC1843 and IRAS 180272). The protocol was approved by these committees. All subjects gave written informed consent in accordance with the Declaration of Helsinki.

## Author Contributions

DA: study design, data collection, data analysis, and manuscript preparation. AA: study design and manuscript preparation. CD: data analysis, data interpretation, and manuscript preparation. DE: study design, data interpretation, and manuscript preparation.

## Conflict of Interest Statement

The authors declare that the research was conducted in the absence of any commercial or financial relationships that could be construed as a potential conflict of interest. The reviewer JC and handling Editor declared their shared affiliation.
