# Supplementary material for: Development and Validation of the Vision-Related Dizziness Questionnaire
Source: Front Neurol. 2018 May 29;9:379. doi: 10.3389/fneur.2018.00379 (PMC5992411; doi:10.3389/fneur.2018.00379)
Supplement: Supplementary file 1 [file Presentation_1.PDF]

UNIVERSITY of BRADFORD
Celebrating 50 years

UNIVERSITY of BRADFORD
School of Optometry & Vision Science

Vision Related Dizziness questionnaire (VRD-25)

Instructions

Please complete the following questions, using a tick as in the example below.

1. **Example: Does your dizziness cause you to have difficulty watching moving traffic or trains and / or crossing roads?** (*please answer for whichever activity causes the most dizziness*)

Never	Occasionally (e.g. 1-2 times a month)	Quite often (e.g. once a week) ✓	Very Often (e.g. 2-6 times per week)	All the time (at least once per day)	Activity not applicable to me

Example: How severe is the dizziness that you experience when you are watching moving traffic or trains and / or crossing roads?

Not at all	Mild – minimal problems caused ✓	Moderate – but I cope	So severe I have reduced doing this	So severe I have stopped doing this	Activity not applicable to me

………

When thinking about any dizziness you have had over the past month……..

1. Does your dizziness cause you to have difficulty watching moving traffic or trains and / or crossing roads? (*please answer for whichever activity causes the most dizziness*)

Never	Occasionally (e.g. 1-2 times a month)	Quite often (e.g. once a week)	Very Often (e.g. 2-6 times per week)	All the time (at least once per day)	Activity not applicable to me

How severe is the dizziness that you experience when you are watching moving traffic or trains and / or crossing roads?

Not at all	Mild – minimal problems caused	Moderate – but I cope	So severe I have reduced doing this	So severe I have stopped doing this	Activity not applicable to me

2. Do you have problems walking alongside a busy road because of your dizziness?

Never	Occasionally (e.g. 1-2 times a month)	Quite often (e.g. once a week)	Very Often (e.g. 2-6 times per week)	All the time (at least once per day)	Activity not applicable to me

How severe is the dizziness that you experience when you are walking alongside a busy road?

Not at all	Mild – minimal problems caused	Moderate – but I cope	So severe I have reduced doing this	So severe I have stopped doing this	Activity not applicable to me

3. Do you have problems when moving around because of your dizziness but are okay when seated?

Never	Occasionally (e.g. 1-2 times a month)	Quite often (e.g. once a week)	Very Often (e.g. 2-6 times per week)	All the time (at least once per day)	Activity not applicable to me

How severe is the dizziness that you experience when you are moving around?

Not at all	Mild – minimal problems caused	Moderate – but I cope	So severe I have reduced doing this	So severe I have stopped doing this	Activity not applicable to me

4. How often is moving around your home difficult due to your dizziness?

Never	Occasionally (e.g. 1-2 times a month)	Quite often (e.g. once a week)	Very Often (e.g. 2-6 times per week)	All the time (at least once per day)	Activity not applicable to me

How severe is the dizziness that you experience when you are moving around your home?

Not at all	Mild – minimal problems caused	Moderate – but I cope	So severe I have reduced doing this	So severe I have stopped doing this	Activity not applicable to me

5. Does walking down the aisle of the supermarket increase your dizziness?

Never	Occasionally (e.g. 1-2 times a month)	Quite often (e.g. once a week)	Very Often (e.g. 2-6 times per week)	All the time (at least once per day)	Activity not applicable to me

How severe is the dizziness that you experience when you are walking down the aisle of the supermarket?

Not at all	Mild – minimal problems caused	Moderate – but I cope	So severe I have reduced doing this	So severe I have stopped doing this	Activity not applicable to me

6. Does your dizziness make it difficult for you to walk on uneven or sloping surfaces?

Never	Occasionally (e.g. 1-2 times a month)	Quite often (e.g. once a week)	Very Often (e.g. 2-6 times per week)	All the time (at least once per day)	Activity not applicable to me

How severe is the dizziness that you experience when you are walking on uneven or sloping surfaces?

Not at all	Mild – minimal problems caused	Moderate – but I cope	So severe I have reduced doing this	So severe I have stopped doing this	Activity not applicable to me

7. Is it difficult for you to walk up or down stairs because of your dizziness?

Never	Occasionally (e.g. 1-2 times a month)	Quite often (e.g. once a week)	Very Often (e.g. 2-6 times per week)	All the time (at least once per day)	Activity not applicable to me

How severe is the dizziness that you experience when you are walking up or down stairs?

Not at all	Mild – minimal problems caused	Moderate – but I cope	So severe I have reduced doing this	So severe I have stopped doing this	Activity not applicable to me

8. Do you have difficulty stepping on to or off an escalator because of your dizziness?

Never	Occasionally (e.g. 1-2 times a month)	Quite often (e.g. once a week)	Very Often (e.g. 2-6 times per week)	All the time (at least once per day)	Activity not applicable to me

How severe is the dizziness that you experience when you are using an escalator?

Not at all	Mild – minimal problems caused	Moderate – but I cope	So severe I have reduced doing this	So severe I have stopped doing this	Activity not applicable to me

9. Does your dizziness interfere with your job or household responsibilities?

Never	Occasionally (e.g. 1-2 times a month)	Quite often (e.g. once a week)	Very Often (e.g. 2-6 times per week)	All the time (at least once per day)	Activity not applicable to me

How severe is the dizziness that you experience when you are doing your job or household responsibilities?

Not at all	Mild – minimal problems caused	Moderate – but I cope	So severe I have reduced doing this	So severe I have stopped doing this	Activity not applicable to me

10. Does your dizziness cause difficulties with hand/eye coordination? *E.g. problems when reaching for a door knob.*

Never	Occasionally (e.g. 1-2 times a month)	Quite often (e.g. once a week)	Very Often (e.g. 2-6 times per week)	All the time (at least once per day)	Activity not applicable to me

How severe is the dizziness that you experience when you are attempting hand/eye coordination?

Not at all	Mild – minimal problems caused	Moderate – but I cope	So severe I have reduced doing this	So severe I have stopped doing this	Activity not applicable to me

11. Is it difficult for you to concentrate because of your dizziness?

Never	Occasionally (e.g. 1-2 times a month)	Quite often (e.g. once a week)	Very Often (e.g. 2-6 times per week)	All the time (at least once per day)	Activity not applicable to me

How severe is the dizziness that you experience when you have difficulty concentrating?

Not at all	Mild – minimal problems caused	Moderate – but I cope	So severe I have reduced doing this	So severe I have stopped doing this	Activity not applicable to me

12. Does your dizziness cause you to feel confused or disorientated?

Never	Occasionally (e.g. 1-2 times a month)	Quite often (e.g. once a week)	Very Often (e.g. 2-6 times per week)	All the time (at least once per day)	Activity not applicable to me

How severe is the dizziness that causes you to feel confused or disorientated?

Not at all	Mild – minimal problems caused	Moderate – but I cope	So severe I have reduced doing this	So severe I have stopped doing this	Activity not applicable to me

13. **Does your dizziness interfere with your ability to enjoy or participate in social activities, sports or pastimes?**

Never	Occasionally (e.g. 1-2 times a month)	Quite often (e.g. once a week)	Very Often (e.g. 2-6 times per week)	All the time (at least once per day)	Activity not applicable to me

How severe is the dizziness that interferes with your ability to enjoy or participate in social activities, sports or pastimes?

Not at all	Mild – minimal problems caused	Moderate – but I cope	So severe I have reduced doing this	So severe I have stopped doing this	Activity not applicable to me

THANK YOU FOR COMPLETING THIS QUESTIONNAIRE
